# A Prospective Crossover Clinical Trial of Esaxerenone and Eplerenone in Patients with Chronic Heart Failure Complicated by Hypertension

**DOI:** 10.3390/life15050741

**Published:** 2025-05-05

**Authors:** Akira Sezai, Msasnori Abe, Takashi Maruyama, Makoto Taoka, Hisakuni Sekino, Masashi Tanaka

**Affiliations:** 1 Department of Cardiovascular Surgery, Nihon University School of Medicine, 30-1 Oyaguchi-kamimachi, Itabashi-ku, Tokyo 173-8610, Japan; taoka.makoto@nihon-u.ac.jp (M.T.); tanaka.masashi@nihon-u.ac.jp (M.T.); 2 Division of Nephrology, Hypertension, and Endocrinology, Department of Medicine, Nihon University School of Medicine, 30-1 Oyaguchi-kamimachi, Itabashi-ku, Tokyo 173-8610, Japan; abe.masanori@nihon-u.ac.jp (M.A.); maruyama.takashi@nihon-u.ac.jp (T.M.); 3 Sekino Hospital, 3-28-3, Ikebukuro, Toshima-ku, Tokyo 171-0014, Japan; obatak@sekino-hospital.com

**Keywords:** heart failure, chronic heart failure, mineralocorticoid receptor antagonist, esaxerenone, eplerenone

## Abstract

Esaxerenone, which blocks aldosterone binding, is approved to treat hypertension but not heart failure. We aimed to understand esaxerenone’s efficacy in treating chronic heart failure. This crossover study compared esaxerenone with eplerenone, an approved treatment for heart failure, in 66 patients with chronic heart failure complicated by hypertension (12 months for each drug). The primary endpoint was brain natriuretic peptide. The secondary endpoints included blood pressure; hormones and enzymes that regulate electrolytes and stress response; and biomarkers of kidney function. Change in brain natriuretic peptide concentration was significantly lower for esaxerenone compared with eplerenone at 3, 6, and 12 months. Blood pressure (all time points), plasma aldosterone concentration (3 and 6 months), and urinary albumin-to-creatinine ratio (3 and 6 months) were significantly lower for esaxerenone compared with eplerenone. The results suggest that esaxerenone more strongly blocks aldosterone binding than does eplerenone. This effect, together with its strong antihypertensive effect and reduced urinary albumin-to-creatinine ratio, suggests that esaxerenone improves kidney function. The results of this small-scale, single-center study need to be expanded to a larger-scale investigation, but esaxerenone shows promise as a treatment for chronic heart failure with hypertension.

## 1. Introduction

The introduction of the “Fantastic four” in the treatment of heart failure has improved the re-admission rate due to heart failure and its prognosis. Meta-analysis studies have recently reported not only cardioprotective effects but also renal protective effects, including an improvement of urinary albumin, and an improvement of right ventricular function [1,2,3].

Aldosterone, released by the adrenal glands as a blood pressure regulator, is a well-known cause of sodium reabsorption, water retention, and potassium and magnesium loss. Aldosterone binds to the mineralocorticoid receptor (MR), causing cardiac, vascular, and kidney organ damage; endothelial and baroreceptor dysfunction; and fibrosis in the myocardium [4]. Mineralocorticoid receptor antagonists (MRAs) are drugs that block the binding of aldosterone to the MR, which disrupts downstream effects and can prevent and treat cardiovascular and kidney diseases [5,6]. Mineralocorticoid receptor antagonists act on MRs in the kidney tubules, inhibiting sodium reabsorption and potassium excretion, and promoting sodium excretion while retaining potassium, resulting in antihypertensive effects. Mineralocorticoid receptor antagonists are also effective against heart failure and chronic kidney disease. In recent years, MRAs have become indispensable for the treatment of heart failure, especially as one of the “Fantastic four” drugs now recommended as the standard treatment for heart failure with reduced ejection fraction (HFrEF) [7].

In patients with chronic heart failure, high blood concentrations of the mineralocorticoid aldosterone and the glucocorticoid cortisol are independent predictors of increased risk of death [8]. The RALES (Randomized Aldactone Evaluation Study) trial evaluated patients with heart failure with New York Heart Association (NYHA) Class III or IV and left ventricular ejection fraction of less than 35% who were on standard treatment for chronic heart failure. This trial tested spironolactone, a first-generation MRA, and found it reduced all-cause mortality, death from heart failure, and hospitalization for heart failure. However, gynecomastia and breast pain were observed in 10% of patients [9]. Subsequently, eplerenone was developed as a second-generation MRA with greater selectivity for the MR than spironolactone and less incidence of gynecomastia. The EPHESUS (Eplerenone Post-Acute Myocardial Infarction Heart Failure Efficacy and Survival Study) trial evaluated patients with acute myocardial infarction with left ventricular ejection fraction of 40% or less with heart failure who were receiving standard therapy for heart failure. This trial tested eplerenone and found it reduced all-cause mortality, death from heart failure, and hospitalization for heart failure [10]. Eplerenone also reduced all-cause mortality, death from heart failure, and hospitalization for heart failure in the EMPHASIS-HF (Eplerenone in Mild Patients Hospitalization and Survival Study in Heart Failure) trial in patients with chronic heart failure with NYHA class II and left ventricular ejection fraction of 35% or less who were receiving standard treatment for heart failure [11].

Esaxerenone, developed as a third-generation MRA, is a nonsteroidal MRA that, like eplerenone, is highly selective for mineralocorticoid receptors and has no steroidal backbone [12]. However, although esaxerenone is approved for antihypertensive therapy, it is not approved to treat heart failure, and its effect on treating chronic heart failure is not clear. In this prospective crossover clinical trial, we compared esaxerenone with eplerenone in patients with chronic heart failure complicated by hypertension. The aim of the study is to clarify the differences between esaxerenone and eplerenone, their respective effects, and any problems.

## 2. Materials and Methods

### 2.1. Study Protocol

The subjects of this study were Japanese patients with hypertension and chronic heart failure and a home morning systolic blood pressure of 140 mmHg or higher. The definition of chronic heart failure in this study was patients with a previous hospitalization for heart failure who were taking at least two standard heart failure medications (angiotensin II receptor blockers, angiotensin-converting enzyme inhibitors, beta-blockers, diuretics, sodium–glucose transport protein 2 inhibitors, or inotropic drugs). Eligible patients were at least 20 years old but less than 90 years old. Exclusion criteria were (a) estimated glomerular filtration rate (eGFR) of less than 30 mL/min/1.73 m^2^ or (b) as judged by the attending physician. The details of the study were explained to each patient, and informed consent was obtained from each patient. The study was conducted in accordance with the Declaration of Helsinki and approved by the Institutional Review Board of Sekino Hospital (protocol no. 20181201) on 2 April 2019. The study was registered with the University Hospital Medical Information Network (http://www.umin.ac.jp/) (study ID: UMIN000037111, Date of registration: 19 July 2019).

We randomized by the envelope method 66 patients to receive treatment with either 2.5 mg esaxerenone (MINNEBRO^®^, Daiichi Sankyo Company, Ltd., Tokyo, Japan) or 50 mg eplerenone (Selara^®^, Viatris Inc., Canonsburg, PA, USA) for 12 months. After 12 months of treatment, patients who were assigned to esaxerenone were switched to eplerenone, while those who were assigned to eplerenone were switched to esaxerenone, and each drug was administered for a further 12 months. For ethical reasons, this study did not include a washout period. When postdose systolic blood pressure was 140 mmHg or higher, we administered a calcium channel blocker or an alpha-blocker, which have less effect on the renin–angiotensin–aldosterone system than do esaxerenone or eplerenone, or the esaxerenone or eplerenone dose was increased. When postprandial systolic blood pressure fell below 90 mmHg or when symptoms such as lightheadedness were reported, patients receiving calcium channel blockers or alpha-blockers had that treatment discontinued or the esaxerenone or eplerenone dose was reduced, and patients not receiving calcium channel blockers or alpha-blockers were given a reduced or no dose of esaxerenone or eplerenone.

### 2.2. Endpoints

The primary endpoint was brain natriuretic peptide (BNP). The secondary endpoints were as follows: (a) home morning blood pressure; (b) the kidney-related biomarkers blood urea nitrogen, serum creatinine, estimated glomerular filtration rate (eGFR); (c) plasma renin activity (PRA), angiotensin II, plasma aldosterone concentration (PAC), cortisol; (d) atrial natriuretic peptide (ANP); (e) sodium and potassium (serum and urinary), urinary sodium-to-potassium ratio (U-Na/K); and (f) urinary albumin-to-creatinine ratio (UACR), urinary collagen IV, urinary β2-microglobulin (U-β2MG), and urinary liver-type fatty acid binding protein (U-L-FABP). Blood samples were taken before the administration of all drugs. Brain natriuretic peptide, ANP, blood urea nitrogen, serum creatinine, eGFR, serum sodium, and serum potassium were measured 1, 3, 6, and 12 months after the start of drug treatment; plasma renin, angiotensin II, plasma aldosterone, UACR, urinary collagen IV, U-β2MG, and U-L-FABP were measured 3, 6, and 12 months after the start of drug treatment; cortisol was measured 6 and 12 months after the start of drug treatment. The changes from before the start of drug administration (baseline) to each time point were evaluated.

### 2.3. Adverse Events

Adverse reactions were classified as kidney dysfunction (increase of creatinine by ≥50%), hepatic dysfunction (increase of aspartate aminotransferase and alanine aminotransferase by ≥50%), and allergic reactions. Management of the adverse reactions was decided by the attending physician. The definition of major adverse cardiovascular and cerebrovascular events in this study was death, ischemic heart disease, cerebrovascular disease, heart failure, or arrhythmia, requiring hospital treatment.

### 2.4. Statistical Analysis

Measured values are expressed as mean ± standard deviation. A *p* value of less than 0.05 was considered statistically significant. Because this crossover study did not include a washout period, we performed mixed-effects model analyses to estimate least-squares means. The model included period, treatment, and sequence as the fixed-effect terms and patient ID as a random effect. All analyses were performed with SPSS software (version 28.0.0.0; IBM Corp., Armonk, NY, USA). Data aggregation was performed by Sekino Laboratory staff who were not involved in this study, and statistical analysis was supported by DataSeed Inc. (Katsushika-ku, Tokyo, Japan), a company not involved in conducting the study.

## 3. Results

### 3.1. Patient Characteristics and Treatment Compliance

We enrolled 66 patients in the study, and their baseline characteristics and blood and urine test results are presented in Table 1 and Table 2. In terms of classifying heart failure, heart failure with reduced ejection fraction (HFrEF) accounted for 3% of patients, heart failure with mildly reduced ejection fraction (HFmrEF) accounted for 2% of patients, and heart failure with preserved ejection fraction (HFpEF) accounted for 95% of patients. In terms of classifying chronic kidney disease, 45% of the patients were in chronic kidney disease stage G3a and 27% were in stage G3b, making up 72% of the total number of patients enrolled in the study.

During the study, the number of patients who received additional or increased doses of the antihypertensive drugs esaxerenone and eplerenone because their blood pressure rose above 140 mmHg was two for esaxerenone (one at 3 months and one at 11 months after the start of drug treatment) and seven for eplerenone (one at 1 and 2 months, four at 3 months, one at 3 and 6 months, and one at 8 months). The number of patients who discontinued or reduced esaxerenone and eplerenone treatment due to systolic blood pressure below 100 mmHg or symptoms such as lightheadedness was three for esaxerenone (one at 1 month, one at 3 months, and one at 4 months) and one for eplerenone (one at 6 months).

### 3.2. Adverse Events

One patient who received eplerenone had their diuretic dosage increased due to worsening heart failure. There were no other adverse cardiovascular events. One patient who received eplerenone discontinued treatment due to gynecomastia. There were no cases of discontinuation due to liver or kidney dysfunction.

### 3.3. Primary Endpoint

**Figure 1:**
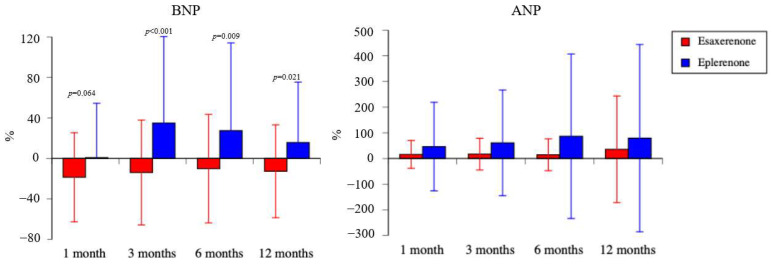
Percentage change for brain natriuretic peptide and atrial natriuretic peptide.

The percentage change data for BNP concentration are summarized in Figure 1. In the esaxerenone group, the percentage change of BNP concentration after the start of drug treatment was −18.6 ± 44.0% at 1 month, −13.9 ± 51.8% at 3 months, −10.1 ± 53.6% at 6 months, and −12.7 ± 45.9% at 12 months. In the eplerenone group, the percentage change of BNP concentration after the start of drug treatment was 0.8 ± 53.7% at 1 month, 35.0 ± 85.4% at 3 months, 27.5 ± 86.6% at 6 months, and 15.7 ± 59.7% at 12 months. Although there was no statistically significant difference between the two groups 1 month after the start of drug treatment (*p* = 0.064), thereafter, the esaxerenone group had significantly lower percentage change of BNP concentration than the eplerenone group (3 months, *p* < 0.001; 6 months, *p* = 0.009; 12 months, *p* = 0.021).

### 3.4. Secondary Endpoints

**Figure 2:**
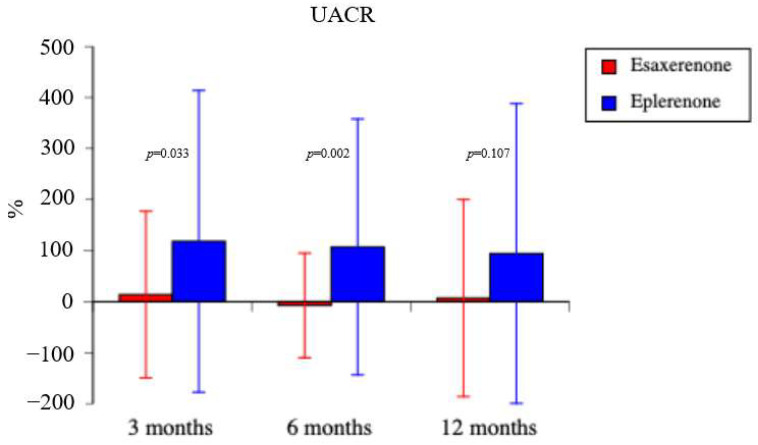
Percentage change for urinary albumin-to-creatinine ratio.

(a) Home morning blood pressure (Table 3): At all time points, the percentage change for both systolic and diastolic blood pressure were statistically significantly lower in the esaxerenone group than in the eplerenone group.

(b) Blood urea nitrogen, serum creatinine, eGFR (Table 3): There were no statistically significant differences between the two groups at any time point for blood urea nitrogen. Serum creatinine was statistically significantly lower in the esaxerenone group than in the eplerenone group 1 month after the start of drug treatment, and eGFR was statistically significantly higher in the esaxerenone group than in the eplerenone group 1 month after the start of drug treatment, but thereafter there were no statistically significant differences between the two groups for either serum creatinine or eGFR.

(c) Plasma renin activity, angiotensin II, PAC, cortisol (Table 3): PRA was statistically significantly higher in the esaxerenone group than in the eplerenone group 12 months after the start of drug treatment, but there were no statistically significant differences between the two groups at the other time points. There were no statistically significant differences between the two groups at any time point for angiotensin II. PAC was statistically significantly higher in the esaxerenone group than in the eplerenone group 3 and 6 months after the start of drug treatment, but there was no statistically significant difference between the two groups at 12 months. There were no statistically significant differences in cortisol between the two groups at all time points. The mean changes from baseline for PRA, angiotensin II, PAC, and cortisol were all positive.

(d) The percentage change data for ANP concentration are summarized in Figure 1. In the esaxerenone group, the percentage change of ANP concentration after the start of drug treatment was 15.8 ± 54.3% at 1 month, 17.1 ± 61.8% at 3 months, 14.5 ± 62.0% at 6 months, and 35.7 ± 207.9% at 12 months. In the eplerenone group, the percentage change of ANP concentration after the start of drug treatment was 46.3 ± 172.7% at 1 month, 60.9 ± 206.0% at 3 months, 86.7 ± 320.6% at 6 months, and 79.3 ± 365.0% at 12 months. There were no statistically significant differences between the two groups at all time points (1 month, *p* = 0.207; 3 months, *p* = 0.143; 6 months, *p* = 0.127; 12 months, *p* = >0.999), and the mean changes from baseline for all these measures were positive.

(e) Sodium and potassium (serum and urinary), U-Na/K (Table 3 and Table 4): there were no statistically significant differences between the two groups for serum and urinary sodium and potassium and U-Na/K at all time points.

(f) UACR (Figure 2); urinary collagen IV, U-β_2_MG, and U-L-FABP (Table 4): In the esaxerenone group, the percentage change of UACR after the start of drug treatment was 13.8 ± 163.4% at 3 months, −7.4 ± 102.5% at 6 months, and 7.0 ± 193.2% at 12 months. In the eplerenone group, the percentage change of UACR after the start of drug treatment was 118.2 ± 295.6% at 3 months, 107.1 ± 250.8% at 6 months, and 94.3 ± 293.7% at 12 months. The UACR was statistically significantly lower in the esaxerenone group than in the eplerenone group at 3 and 6 months after the start of drug treatment (3 months, *p* = 0.033; 6 months, *p* = 0.002) but not at 12 months (*p* = 0.107). Urinary collagen IV was statistically significantly lower in the esaxerenone group than in the eplerenone group 6 and 12 months after the start of drug treatment, but there was no statistically significant difference between the two groups at 3 months. There were no statistically significant differences between the two groups for U-β_2_MG and U-L-FABP at any time point.

## 4. Discussion

Our results show that esaxerenone has a stronger antihypertensive effect and causes a greater reduction in BNP concentration than eplerenone in patients with hypertension and chronic heart failure. These results arise because esaxerenone has a stronger effect on blocking aldosterone than does eplerenone. The resultant positive effect on the kidneys may explain why the percentage change of UACR was lower in patients treated with esaxerenone compared with those treated with eplerenone.

In this study, we compared the antihypertensive effects of 2.5 mg esaxerenone and 50 mg eplerenone. In a phase 3 clinical trial of esaxerenone, the percentage change in systolic blood pressure was −13.7 mmHg for 2.5 mg esaxerenone, −16.9 mmHg for 5 mg esaxerenone, and −12.1 mmHg for 50 mg eplerenone. There was no significant difference between 2.5 mg esaxerenone and 50 mg eplerenone (*p* = 0.0709), and there was a significant difference between 5 mg esaxerenone and 50 mg eplerenone (*p* < 0.001) [13]. Furthermore, a post hoc analysis of 24 h mean ambulatory blood pressure from a phase 3 study of esaxerenone showed that the reduction in blood pressure at night with esaxerenone was stronger than with eplerenone and that it was particularly effective in elderly patients and patients with a non-dipper pattern of night-time blood pressure [14]. We found 2.5 mg esaxerenone was significantly more effective in lowering blood pressure than 50 mg eplerenone because (a) our study measured home morning blood pressure, whereas the phase 3 study used blood pressure measured during a medical examination, (b) the mean ± standard deviation age of the patients in our study was 72.3 ± 9.7 years, whereas the mean ± standard deviation age of the patients in the phase 3 study was 55.5 ± 9.6 years, (c) our study was a study of chronic heart failure, and the background of the patients was different, and (d) the half-life of eplerenone is shorter than that of esaxerenone (esaxerenone 5 mg: 18.6 ± 2.38 h, eplerenone 100 mg: 3.0 ± 0.7 h, per Interview Form of both drugs).

In our study, the percentage change for PAC and PRA increased after administration of either esaxerenone or eplerenone, with significantly greater increases with esaxerenone than with eplerenone at some time points, the same pattern as seen in the phase 3 study of esaxerenone [13]. The study authors suggest the antihypertensive effect of esaxerenone occurs by inhibiting the aldosterone receptor: “esaxerenone may act more on kidney tubules and inhibit MR in other tissues more strongly than eplerenone” [13]. Generally, high blood aldosterone concentration contributes to organ damage, but when an MRA is administered, aldosterone concentrations increase, which is thought to indicate that MRAs block aldosterone receptors. In a comparative study of the angiotensin II receptor blockers olmesartan, candesartan, and azilsartan, olmesartan suppressed aldosterone, which in turn suppressed weight of the heart muscle and reduced BNP [15,16]. The authors concluded that it is important to suppress the effects of aldosterone in patients with cardiovascular disease.

Regarding the kidney-protective effects of MRAs, our results show that both drugs caused a mild deterioration in serum creatinine concentration and eGFR compared with before drug administration, but these measures improved over time; esaxerenone showed significantly better results than eplerenone immediately after the start of drug administration and at 12 months. Eplerenone treatment increased UACR, whereas esaxerenone decreased UACR at some time points, and the decrease was significantly greater than that of eplerenone. Urinary sodium and urinary sodium/potassium concentrations increased after treatment with either drug, but there was no difference for urinary sodium/potassium concentrations in the patients treated with either drug. In animal experiments, aldosterone induces inflammation, activates MRs, and promotes kidney fibrosis; this pathology is inhibited by esaxerenone, decreasing UACR and suppressing kidney fibrosis [12,17]. An analysis of the phase 3 study of esaxerenone showed that esaxerenone directly lowers UACR independent of its blood pressure-lowering effect, and that this effect is much greater than the blood pressure-dependent effect [18]. Therefore, esaxerenone could be used to lower UACR in patients with diabetic nephropathy. Ito et al. conducted a clinical study in which esaxerenone was added to the treatment of 56 patients with type 2 diabetes and UACR ≥ 300 mg/g who were taking a RAS (renin–angiotensin system) inhibitor. Esaxerenone treatment changed the eGFR by −8.3 mL/min/1.73 m^2^ from baseline but caused a 54.8% decrease in UACR. The proportion of patients with a 30% or greater reduction in UACR was high at 75.0%, and improvement in early nephropathy was observed in 51.8% of patients. The decline in eGFR did not persist after treatment was discontinued, and eGFR recovered to baseline by 4 weeks after the end of the study, within a clinically acceptable range [19]. In a study of patients with hypertension with moderate kidney impairment, eGFR decreased up to 12 weeks after the start of drug administration but then recovered to baseline. A transient decline in eGFR has also been reported for all MRAs; “this decline is thought to be due to the improvement in kidney glomerular hyperfiltration” [20,21,22]. In our study, esaxerenone significantly reduced UACR compared with eplerenone, but the reduction was lower than in previous reports. We believe this discrepancy can be explained because the previous reports were based on patients with overt albuminuria (≥300 mg/g creatinine) before drug administration, while in our study, 33 cases (48.5%) had microalbuminuria (30–299 mg/g creatinine) before drug administration and only 3 cases (4.5%) had macroalbuminuria.

We found no difference in urinary sodium concentration in patients treated with esaxerenone or with eplerenone, but both drugs increased urinary sodium concentration in both patient groups. In addition, urinary sodium/potassium concentration also increased in both patient groups. Mineralocorticoid receptor antagonists inhibit sodium reabsorption and potassium excretion. In animal experiments, esaxerenone dose-dependently inhibits the decrease in U-Na/K caused by aldosterone administration [23]. Itoh et al. measured urinary markers of nephropathy after treatment with esaxerenone, including 8-hydroxydeoxyguanosine, angiotensinogen, β_2_-MG, L-FABP, and N-acetyl-β-d-glucosaminidase. Only β_2_-MG decreased with esaxerenone administration [24]. In our study, there was no difference between the two drugs for β_2_-MG, U-L-FABP, and N-acetyl-β-d-glucosaminidase, but urinary collagen IV, an earlier measure of glomerular filtration dysfunction than UACR, decreased significantly after esaxerenone treatment but not after eplerenone treatment.

Regarding the circulating cardiac hormones BNP and ANP, we found that esaxerenone reduced BNP but eplerenone did not, and ANP increased after MRA administration. There are many reports of changes in BNP or N-terminal pro-BNP (NT-proBNP) after MRA treatment, but there are almost no reports on changes in ANP after MRA treatment. In an analysis of the results of a phase 3 study of esaxerenone, NT-proBNP concentrations were significantly reduced in all patients with dipping blood pressure patterns due to esaxerenone administration, and the authors concluded that “the myocardial load was reduced due to the decrease in nighttime BP [blood pressure] resulting from the mineralocorticoid receptor antagonism” [25]. In a multicenter, randomized, double-blind, placebo-controlled trial (J-EMPHASIS-HF) of eplerenone in Japanese patients with chronic heart failure (HFrEF), BNP was significantly higher in the eplerenone group than in the placebo group [26]. Atrial natriuretic peptide is a hormone involved in sodium diuresis. A recombinant form of human ANP, carperitide, decreases blood BNP concentration, increases ANP concentration, suppresses the renin–angiotensin–aldosterone system, and has cardioprotective and renoprotective effects through sodium diuresis [27,28,29]. In a RALES study, the starting dose of Spironolactone at 12.5 mg significantly decreased N-terminal proatrial natriuretic factor (pro-ANF) compared to the placebo, suggesting beneficial effects on atrial pressure. It is known that pro-ANF is related to the right atrial and pulmonary capillary wedge pressure and is an important predictive factor for survival in patients with heart failure [9,30]. Ichikawa et al. reported that esaxerenone administration increased PAC and PRA and decreased ANP and NT-proBNP concentrations and urine volume and sodium. They inferred that esaxerenone transiently increases sodium excretion for up to two weeks after administration and that the excess sodium is sufficiently excreted from the body, subsequently decreasing urinary sodium excretion. However, they reported that their finding was a limitation of the study because there were no data on urinary sodium excretion within two weeks of esaxerenone administration [31]. Their study included patients with normal ANP and NT-proBNP concentrations and no complications related to kidney disease or heart failure. In a study by Yano et al. that administered eplerenone to patients with hypertension taking RAS inhibitors, both ANP and BNP decreased after the administration of eplerenone, and there was a significant relationship between the decrease in night-time blood pressure and ANP and BNP concentrations. As reasons for the decrease in ANP and BNP, the authors considered that “first, the BP [blood pressure] reduction realized by eplerenone treatment may have been at least partly due to a reduction in intravascular volume; second, the reduction of ANP or BNP concentrations may have been merely a marker of the reduction of cardiac afterload (i.e., pressure load) by the BP-lowering effect of eplerenone” [32]. In contrast, all our research subjects were patients with chronic heart failure, and approximately 70% of them had chronic kidney disease. Our results, which show that urinary sodium increases when ANP concentrations are high, and the results of Ichikawa et al. [31], which show that urinary sodium decreases when ANP concentrations are low, indicate that ANP is strongly involved in the natriuretic effect. However, the direct effect of esaxerenone on ANP cannot be clearly determined due to the different patient groups involved, and the effect of MRAs on ANP remains to be clarified. More research is needed for MRA and ANP.

Although there are many reports on the effectiveness of spironolactone and eplerenone in patients with heart failure, there are only a few studies on esaxerenone in patients with heart failure, such as our study. Imamura et al. conducted a small-scale clinical study using esaxerenone alone; they administered esaxerenone to 33 patients with chronic heart failure (HFpEF) complicated by hypertension and reported that esaxerenone significantly reduced systolic blood pressure, left ventricular weight index, and BNP concentrations and may have a beneficial effect on reverse remodeling in patients with HFpEF [33]. Naruke et al. administered esaxerenone to 48 patients with chronic heart failure complicated by hypertension and reported that systolic blood pressure and BNP concentrations decreased significantly [34]. The majority of patients in our study had chronic heart failure with HFpEF. Hypertension is often involved in the cause of HFpEF, and a meta-analysis of 41,972 patients with chronic heart failure reported that hypertension was present in 51% of cases of HFpEF, a higher proportion than in cases of HFrEF [35]. In Japan, hypertension is a common cause of HFpEF; in the JCARE-CARD study, it was the most common cause at 44% of cases with HFpEF [36], and in the CHART-2 study, it was the second most common cause after ischemic heart disease at 24.5% of cases with HFpEF [37]. Our results suggest that esaxerenone may be more effective than eplerenone for treating HFpEF. We believe that esaxerenone should be used in the future for treating cases of HFpEF with hypertension. However, our study was small-scale and conducted at a single facility, and the occurrence of cardiovascular events was not examined.

In a large-scale study (FINEARTS-HF trial) of finerenone, a non-steroidal MRA similar to esaxerenone, conducted in patients with HFmrEF and HFpEF, finerenone significantly reduced the composite incidence of total events due to worsening heart failure and cardiovascular death compared to placebo [38]. Furthermore, a meta-analysis of MRAs—including steroidal MRAs such as spironolactone and esaxerenone, and the non-steroidal MRA finerenone—showed that steroidal MRAs reduced cardiovascular death and heart failure hospitalization in patients with HFrEF, while non-steroidal MRAs were effective in reducing risks in patients with HFmrEF and HFpEF [39]. Although no large-scale study of esaxerenone in heart failure has been conducted to date, it remains of great interest to determine whether it demonstrates efficacy comparable to that of finerenone. We therefore hope to conduct comparative studies between esaxerenone and finerenone in the future.

This study has several limitations. It was a small-scale, single-center study. We performed a crossover study, but there was no washout period so as to avoid deterioration of patient health. Most of the cases had HFpEF, but there were also cases of HFrEF and HFmrEF. The dose of esaxerenone was limited to 2.5 mg.

Our study suggests that although esaxerenone and eplerenone are both MRAs, they may differ in their mechanisms of action, antihypertensive effects, and strength of MR blocking. However, the scope of the study did not include clarifying the differences in the mechanisms of action. Here, based on our ANP results, we suggest for the first time that MRAs may have some effect on natriuretic peptides. Furthermore, at the time when this study was conducted, SGLT2 inhibitors which have been shown to be effective in HFrEF as well as HFpEF were not yet approved in Japan. Only 12 percent of patients in our study had received SGLT2 inhibitors. This is considered as a limitation of this study.

Esaxerenone will need to be examined in large-scale studies, but we expect it to be a promising treatment option, especially for patients with heart failure complicated by hypertension, as one of the “fantastic four” drugs.

## 5. Conclusions

Esaxerenone likely has a stronger effect on blocking aldosterone than does eplerenone. We think that the strong antihypertensive effect and the effect of blocking aldosterone has a positive effect on kidney function, explaining why our UACR results were better for esaxerenone than for eplerenone. We believe that further investigation is necessary for understanding the mechanisms of action of these drugs and their potential for clinical use.

## Figures and Tables

**Table 1:** Patient baseline characteristics.

Characteristic	Data Value
Cases, No.	66
Age, years, mean ± SD (range)	72.3 ± 9.7 (38–89)
Gender, male/female, No.	51:15
Classification of heart failure, No. (%)	
HFrEF	2 (3)
HFmrEF	1 (2)
HFpEF	63 (95)
Cause of heart failure, No. (%)	
Ischemic heart disease	17 (26)
Valve disease	31 (47)
Hypertension	15 (23)
Cardiomyopathy	1 (2)
Arrhythmia	2 (3)
Complication, No. (%)	
Diabetes mellitus	30 (45)
Dyslipidemia	61 (92)
Hyperuricemia	29 (44)
CKD (stage G3a)	30 (45)
CKD (stage G3b)	18 (27)
Obesity	16 (24)
Cerebral infarct	6 (9)
Oral medicine, No. (%)	
ACE-I	2 (3)
ARB	44 (67)
Beta-blocker	55 (83)
SGLT2 inhibitor	8 (12)
Diuretic	15 (23)
Digoxin	2 (3)
Pimobendan	1 (1)
Calcium channel blocker	31 (47)
Alpha-blocker	4 (6)

ACE-I: angiotensin-converting enzyme inhibitor, ARB: angiotensin II receptor blocker, CKD: chronic kidney disease, HFmrEF: heart failure with mildly reduced ejection fraction, HFpEF: heart failure with preserved ejection fraction, HFrEF: heart failure with reduced ejection fraction, No.: number, SD: standard deviation, SGLT2: sodium–glucose transport protein 2.

**Table 2:** Pre-study blood and urine test data of patients.

Systolic BP (mmHg)	144.5 ± 7.2
Diastolic BP (mmHg)	78.2 ± 12.0
BNP (pg/mL)	166.3 ± 283.0
ANP (pg/mL)	87.7 ± 58.9
Blood urea nitrogen (mg/dL)	19.4 ± 5.7
Serum creatinine (mg/dL)	1.11 ± 0.33
eGFR (mL/min/1.73 m^2^)	51.6 ± 14.4
Serum sodium (mEq/L)	140.8 ± 2.6
Serum potassium (mEq/L)	4.22 ± 0.43
PRA (ng/mL/h)	2.58 ± 4.76
Angiotensin-II (U/L)	30.3 ± 117.8
PAC (pg/mL)	109.5 ± 62.0
Cortisol (µg/dL)	10.9 ± 3.4
UACR (g/gCr)	94.7 ± 141.7
Urinary sodium (mEq/L)	91.1 ± 38.4
Urinary potassium (mEq/L)	35.5 ± 21.5
Urinary sodium/potassium ratio	3.39 ± 1.86
β_2_-MG (µg/L)	704.8 ± 1150.9
L-FABP (µg/g Cr)	8.85 ± 14.3

BP: blood pressure, BNP: brain natriuretic peptide, ANP: atrial natriuretic peptide, eGFR: estimated glomerular filtration rate, PRA: plasma renin activity, PAC: plasma aldosterone concentration, UACR: urinary albumin-to-creatinine ratio, MG: macroglobulin, L-FABP: liver-type fatty acid binding protein.

**Table 3:** Percentage change for each blood test.

Test	Drug and *p* Value	1 Month	3 Months	6 Months	12 Months
Systolic BP	Esaxerenone	−12.5 ± 12.8	−12.3 ± 12.9	−12.1 ± 12.7	−12.3 ± 12.4
	Eplerenone	−2.6 ± 9.6	−2.2 ± 10.4	−3.1 ± 10.1	−1.9 ± 9.0
	*p* value	<0.001	<0.001	<0.001	<0.001
Diastolic BP	Esaxerenone	−5.4 ± 5.4	−5.2 ± 4.5	−5.9 ± 8.0	−4.4 ± 7.7
	Eplerenone	5.5 ± 6.1	5.4 ± 8.8	5.7 ± 9.0	5.3 ± 6.8
	*p* value	<0.001	<0.001	<0.001	<0.001
BUN	Esaxerenone	11.5 ± 25.1	12.6 ± 28.3	12.4 ± 24.8	10.1 ± 28.6
	Eplerenone	10.3 ± 25.6	10.8 ± 34.4	11.4 ± 33.5	7.1 ± 29.5
	*p* value	0.898	0.898	>0.999	0.832
Serum creatinine	Esaxerenone	0.1 ± 10.9	3.5 ± 16.6	4.1 ± 14.4	1.4 ± 17.1
	Eplerenone	5.4 ± 9.9	6.0 ± 12.4	6.7 ± 14.2	4.6 ± 14.4
	*p* value	0.003	1	0.372	0.221
eGFR	Esaxerenone	0.14 ± 10.6	−1.9 ± 16.8	−3.1 ± 17.1	0.26 ± 18.4
	Eplerenone	−5.2 ± 10.3	−6.0 ± 12.8	−5.9 ± 13.1	−3.7 ± 15.0
	*p* value	0.002	0.134	0.344	0.116
Serum sodium	Esaxerenone	−0.41 ± 1.72	−0.35 ± 2.16	−0.29 ± 1.97	−0.03 ± 1.76
	Eplerenone	−0.13 ± 1.32	0.22 ± 1.67	0.18 ± 1.57	−0.26 ± 2.53
	*p* value	0.308	0.111	0.093	0.593
Serum potassium	Esaxerenone	4.6 ± 10.2	5.8 ± 12.2	5.4 ± 12.2	4.0 ± 10.4
	Eplerenone	3.1 ± 8.1	2.9 ± 10.5	3.0 ± 11.2	1.9 ± 10.3
	*p* value	0.521	0.251	0.322	0.501
PRA	Esaxerenone	-	194.0 ± 49.6	285.7 ± 84.1	349.9 ± 66.9
	Eplerenone	-	126.0 ± 48.7	166.1 ± 82.6	106.1 ± 66.6
	*p* value	-	0.334	0.316	0.012
Angiotensin II	Esaxerenone	-	194.9 ± 88.1	139.4 ± 75.9	174.8 ± 69.6
	Eplerenone	-	109.5 ± 50.1	83.7 ± 45.1	59.7 ± 48.7
	*p* value	-	0.299	0.392	0.104
PAC	Esaxerenone	-	58.0 ± 14.7	66.9 ± 24.0	42.9 ± 25.5
	Eplerenone	-	21.2 ± 14.7	6.6 ± 23.6	14.3 ± 25.1
	*p* value	-	0.042	0.039	0.115
Cortisol	Esaxerenone	-	8.4 ± 33.2	8.6 ± 32.4	8.6 ± 35.0
	Eplerenone	-	10.2 ± 39.8	10.4 ± 40.9	12.3 ± 35.6
	*p* value	-	0.722	0.639	0.646

BP: blood pressure, BUN: urea nitrogen, eGFR: estimated glomerular filtration rate, PAC: plasma aldosterone concentration, PRA: plasma renin activity.

**Table 4:** Percentage change for each urine test.

Test	Drug and *p* Value	3 Months	6 Months	12 Months
Sodium	Esaxerenone	25.2 ± 82.3	10.8 ± 56.2	19.7 ± 81.3
	Eplerenone	47.5 ± 227.9	21.3 ± 101.8	40.5 ± 211.7
	*p* value	0.419	0.301	0.382
Potassium	Esaxerenone	97.8 ± 308.3	76.8 ± 387.8	82.7 ± 336.5
	Eplerenone	35.6 ± 148.5	33.3 ± 131.3	24.4 ± 131.9
	*p* value	1	0.561	0.278
Urinary sodium/potassium ratio	Esaxerenone	21.3 ± 95.0	16.9 ± 84.8	26.2 ± 97.2
	Eplerenone	89.6 ± 472.5	31.9 ± 108.1	40.9 ± 109.7
	*p* value	0.995	0.287	1
Collagen IV	Esaxerenone	67.7 ± 505.5	−2.6 ± 53.7	−1.7 ± 62.8
	Eplerenone	45.6 ± 90.5	34.5 ± 100.0	39.0 ± 79.3
	*p* value	0.771	0.013	0.042
β_2_-MG	Esaxerenone	157.5 ± 124.3	81.7 ± 32.1	263.9 ± 87.0
	Eplerenone	314.5 ± 122.1	130.1 ± 30.8	130.3 ± 85.0
	*p* value	0.374	0.230	0.280
L-FABP	Esaxerenone	61.4 ± 73.7	64.0 ± 145.1	53.4 ± 149.2
	Eplerenone	172.9 ± 77.7	101.9 ± 35.1	15.6 ± 39.3
	*p* value	0.326	0.458	>0.999

L-FABP: liver-type fatty acid binding protein, MG: macroglobulin.

## Data Availability

The data presented in this study are available on request from the corresponding author due to privacy restrictions (the data contain information that could compromise the privacy of the research participants).
